# Asking what matters: The relevance and use of patient‐reported outcome measures that were developed without patient involvement

**DOI:** 10.1111/hex.12573

**Published:** 2017-07-04

**Authors:** Bianca Wiering, Dolf de Boer, Diana Delnoij

**Affiliations:** ^1^ Tranzo (Scientific Centre for Transformation in Care and Welfare) Tilburg University Tilburg The Netherlands; ^2^ NIVEL (Netherlands Institute for Health Services Research) Utrecht The Netherlands

**Keywords:** hip and knee surgery, importance ratings, patient preferences, patient‐reported outcome measures

## Abstract

**Background:**

Patient‐reported outcome measures (PROMs) are increasingly used to establish the value of health care. In order to reflect value, PROMs should measure outcomes that matter to patients. However, patients are not always involved in the development of PROMs. This study therefore aimed to investigate whether PROMs, which were developed without patient involvement, are relevant to patients and whether the level of importance allocated towards aspects of these PROMs varies between patient groups.

**Methods:**

All patients from 20 Dutch hospitals undergoing hip or knee surgery in 2014 were invited to a PROMs survey. Participants were asked to rate the importance of each of the items in the HOOS‐Physical Function Short form or the KOOS‐Physical Function Short form, the EQ‐5D and the NRS pain.

**Results:**

Most outcomes were considered important. However, 77.7% of hip surgery patients rated being able to run as unimportant. Being able to kneel (32.7%) or squat (39.6%) was not important to a considerable minority of knee surgery patients. Pain, especially during rest, was considered very important by both hip (68.2%) and knee (66.5%) surgery patients. Patients who were older, male, experienced overall bad health and psychological health considered many items from the PROMs less important than other patients.

**Discussion:**

Patients differ in what they consider important. Health‐care professionals should explore patients’ preferences and discuss which treatment options best fit patients’ preferences. Additionally, if PROMs are used in performance measurement, further research is needed to look at whether and how variation in patient preferences can be taken into account.

## 1 INTRODUCTION

In several countries such as the United States, the United Kingdom, Sweden and the Netherlands, policymakers try to deal with the ever higher spending on health care for mediocre quality of care. They do so by shifting the focus from contracting or paying for health care based on numbers and price towards contracting based on quality.^1^, ^2^, ^3^, ^4^, ^5^, ^6^, ^7^, ^8^ The goal is to achieve the highest value, which is the best possible health outcomes per monetary unit spent.^9^ Delivering good quality of care and thereby achieving good health is less expensive than having to deal with poor health.^10^ Therefore, a value based health‐care system is expected to increase the economic sustainability while benefiting patients, health‐care purchasers and health‐care providers by improving care.^11^ A way to establish value is to measure and compare patient outcomes^12^ and weigh these against treatment costs.^5^

Patient outcomes are increasingly measured using patient‐reported outcome measures (PROMs).^3^, ^4^, ^13^, ^14^ PROMs use the patient as a source of information on health outcomes such as quality of life.^3^, ^15^ Including patient‐reported outcomes is important, as some questions about health care can only be answered by patients.^16^ Additionally, patients offer a different view on outcomes,^17^ and patients are becoming important stakeholders in health care.^18^, ^19^ However, if PROMs are to be used to establish the value of health care, there are two aspects which may need further consideration.

Firstly, research suggests that publicizing health‐care performance results leads to a focus on low scoring aspects of performance in quality improvement efforts.^20^ At first glance this would be something to encourage. However, in the case of PROMs this assumption would only be true if the PROMs are a good reflection of what patients regard as important. In other words, patients should be involved throughout the development, ensuring that PROMs truly reflect the patient's perspective. However, a scoping review of 193 PROMs, including several PROMs which were used in this study, suggests that patients are not always involved in PROM development.^21^ Consequently, health‐care providers may have improved on some aspects of care, but at the same time may have neglected to improve on other aspects of care which are important to patients. Patient outcomes are included because delivering high value for patients should be the main goal of health‐care delivery.^10^, ^11^ Therefore, failing to improve on aspects of care which are important to patients negates any value PROMs may add.

The second aspect of using PROMs that needs further consideration is that, even if PROMs reflect the patients’ perspective, they still only reflect the overall patient population. Usually only the aspects which are considered important by most patients are included, which means that more uncommon symptoms or complaints are neglected. The focus in health care is shifting towards a more person‐centred approach,^22^ whereby the patients are actively involved in their care and care is individualized by recognizing that a patient is a person with specific needs, preferences and values.^8^ This is also relevant for the use of PROMs, as individual patients may differ in the importance they attach to different outcomes.^12^, ^23^ For example, an 80‐year‐old patient living in a nursing home may be less interested in being able to perform physically demanding functions such as running than an active 60‐year‐old patient. Measuring and interpreting health outcomes as if patients regard the measures as equally important may not give an accurate view of how patients perceive the quality of their care.

However, before any methods that take individual differences into account are included, it is important to establish whether PROMs reflect the issues that are important to most patients. There are several types of preference based measures which may give more insight into these issues. Examples are standard gamble, time‐trade‐off and rating scales.^24^, ^25^ Standard gamble and time‐trade‐off ask patients to consider what they would be willing to sacrifice to avoid being in a particular health state.^24^ However, it is our aim to explore the importance patients allocate towards aspects of existing PROMs which were developed without patient involvement. Therefore, we used importance rating scales. Importance ratings are an easy way to look at whether patients regard any part of a PROM as important^26^ and whether there are any differences between what patients value. It allows patients to rate each item separately. It also enables patients to consider something important which may not be worth the trade‐off needed for standard gamble of time‐trade‐off.

To give more insight into whether PROMs, which were developed without patient involvement, can still reflect what patients consider important and if there are any differences in preferences between patients, we used a specific case. Patients undergoing hip or knee surgery were invited to fill in PROMs and rate the importance of the items of the PROMs. By adding importance ratings to the PROMs, we aimed to investigate the following:

1. What is the level of importance patients allocate towards the different aspects of a few well‐known PROMs?
2. Do the levels of importance allocated towards the different aspects of PROMs differ between groups of patients?

## 2 METHODS

### 2.1 Participants

This study is part of a study carried out by a Dutch health insurers collaboration.^27^ All patients in the Netherlands who underwent hip or knee surgery in 2014 were invited by either their health insurer or their hospital, depending on the hospital's choice of data collection (either coordinated via the health insurers or organized by the hospital itself). For 20 hospitals, patients were asked to complete importance rating scales in addition to the standard questionnaire containing patient‐reported experience measures (PREMs) and PROMs. These 20 hospitals were selected based on a high and more or less even number of patients undergoing hip and knee surgery. Hospitals who coordinated their own data collection instead of participating in the data collection via the health insurers were excluded. Patients younger than 16 years and patients who had been invited earlier that year to complete a similar questionnaire were excluded.

### 2.2 Procedure

In the Netherlands, health insurers are legally allowed to contact their clients for participation in research which may help to improve the quality of care. Of course, in doing so they should guarantee the privacy of the patient in all circumstances. An important aspect of the research health insurers did was setting out PREMs and PROMs for certain types of interventions among their clients.

The health insurers sent their clients a letter within 12 months after surgery inviting them to fill in a questionnaire regarding the care they had received. The letter contained a link to the website with the questionnaire and login details. The letter was accompanied by a card which the client could send back if he or she declined to participate. A reminder was sent a week after the invitation letter. A second reminder accompanied by a paper version of the questionnaire was sent 2 weeks later. Three weeks after the paper questionnaire was sent, a fourth reminder was sent.

### 2.3 Measures

The questionnaire was among others comprised of basic information, PROMs, rating scales and a question regarding the main reason for surgery. The basic information used for this study concerned age, sex, education level, overall health, overall psychological health and complications.

Rating scales were added to the HOOS‐Physical Function Short form (HOOS‐PS)^28^ or the KOOS‐Physical Function Short form (KOOS‐PS),^29^ the EQ‐5D^30^ and the NRS pain.^31^ Participants were asked for each of the PROM items how important this item is to them. For example: “How important is being able to descend stairs to you?” Participants could answer on a four‐point scale (1 “Absolutely not important”‐4 “Of the greatest importance”).

The PROMs were developed without patient involvement. Participants completed the HOOS‐PS or the KOOS‐PS depending on whether participants were operated on their hip or knee. The KOOS is based on the WOMAC Osteoarthritis Index, a literature review, an expert panel and a pilot study.^32^ The HOOS is an adaptation of the KOOS.^33^ The HOOS‐PS and KOOS‐PS were created by shortening the HOOS and the KOOS using Rasch analysis.^28^, ^29^ Both questionnaire are validated extensively.^34^, ^35^, ^36^, ^37^ The HOOS‐PS^28^ and the KOOS‐PS^29^ measure physical functioning level. Participants rated the degree of difficulty that was experienced during the last month and the month before surgery due to the hip or knee problems on a five‐point scale (“None”‐ “Extreme”). The HOOS‐PS consists of five items and the KOOS‐PS of seven items.

The EQ‐5D was developed by the EuroQol Group and is validated in many settings worldwide.^30^ The questionnaire measures health status by asking participants to indicate the degree of difficulty participants experienced (“No problems”‐”Major problems”) over five dimensions for the day they completed the questionnaire and just before surgery.

The NRS pain^31^ is a validated numerical rating scale where participants rate their pain intensity from 0 to 10 (0 “No pain”‐10 “Worst possible pain”). Participants rated their pain intensity during rest and while using their hip or knee for the month before surgery and the month preceding the day they completed the questionnaire.

Finally, patients were asked a question regarding their main reason for undergoing surgery: “We can imagine that it is difficult to choose one reason. However, we would like to ask you what your main reason for undergoing hip or knee surgery was?” Answer options were as follows: “Mostly to improve function,” “Mostly to lessen the pain” and “I cannot choose.”

### 2.4 Statistical analyses

Univariate analyses were performed to describe the participant characteristics and to give insight into how participants answered their PROM questions and rating scales. To investigate whether different patients allocated different levels of importance towards certain items, a series of linear regression analyses were performed. The importance ratings were included as dependent variables in separate regression analyses. The analyses were controlled for medical complications and time between surgery and questionnaire completion, as the time between surgery and questionnaire completion varied greatly. The outcome item corresponding to the importance rating (which was used as dependent variable), age, sex, overall health, overall psychological health, educational attainment (university, higher vocational education, middle vocational education, high school/secondary education, <high school level) and main reason for surgery (pain, functioning, cannot choose) were included as independent variables. For analyses regarding PROM items which were answered by all participants, the variable type of surgery (hip or knee) was added. To take into account the high number of regression analyses, a cut‐off point of .01 for the *P* value was used. Analyses were conducted using spss 22.0.^38^

## 3 RESULTS

### 3.1 Response

**Figure 1:**
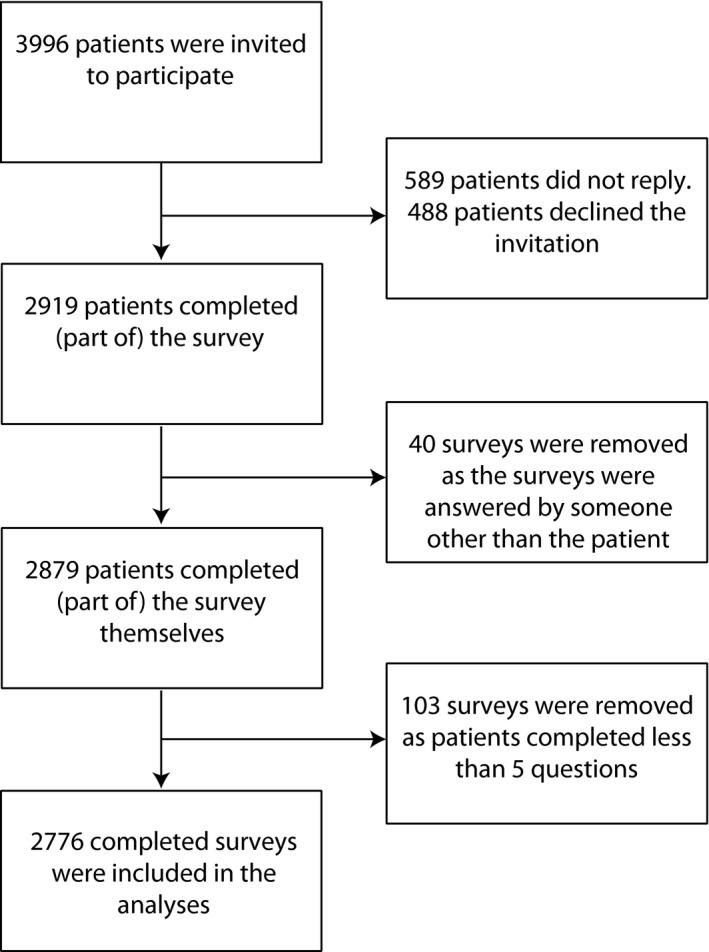
Flow chart

A total of 3996 patients from 20 hospitals were invited to fill in the questionnaire which included importance ratings. 1108 patients participated by filling in the questionnaire online, while 1811 patients used the paper version. A total of 589 patients did not react, while 488 patients declined to participate. This is a response rate of 73.1%. This response rate is slightly higher than the response rate of the bigger study, which was 72.0%. Data from 40 patients were removed as these respondents indicated that someone else answered the questions. Data from 103 patients were removed as these patients completed less than five questions. The final number of included patients was 2776 (69.5%) (Figure 1), which is slightly higher than the bigger study (63.1%). Non‐respondents differed only in age from respondents (73.2 compared to 72.0 years; (*F*(1, 3994)=11.77, *P*=.00).

### 3.2 Sample characteristics

Most participants were female (65.7%) and received secondary education (56.0%) (Table 1). Their average age was 72.0 (range=28‐98; SD=9.1) and a small majority of the participants underwent hip surgery (52.5%). Although 27.0 percent of the participants experienced complications, on average patients improved on all PROMs (Table 1).

**Table 1:** Patient characteristics and PROM scores (N=2776)

	N (%)	Mean (SD)
Age	2776 (100.0%)	72.0 (9.1)
Number of days after surgery	2775 (100.0%)	274.4 (70.2)
HOOS‐PS baseline	1091 (74.8%)	57.0 (22.3)
HOOS‐PS post‐surgery	1104 (75.7%)	25.2 (20.1)
KOOS‐PS baseline	1103 (83.7%)	54.3 (21.4)
KOOS‐PS post‐surgery	1142 (86.6%)	34.5 (18.3)
EQ5D baseline^a^	2107 (75.9%)	0.4 (0.3)
EQ5D post‐surgery^a^	2129 (76.7%)	0.8 (0.2)
NRS pain during rest baseline	2539 (91.5%)	7 (2.6)
NRS pain during rest post‐visit	2595 (93.5%)	2.5 (2.9)
NRS pain during use baseline	2547 (91.8%)	7.9 (2.3)
NRS pain during use post‐surgery	2578 (92.9%)	3 (3)
Sex (Female)	1824 (65.7%)	
Complications (Yes)	750 (27.0%)	
Hip surgery	1458 (52.5%)	
Knee surgery	1318 (47.5%)	
Reason for surgery		
Less pain	966 (34.8%)	
Improved functioning	1212 (43.7%)	
Unable to choose between reasons	272 (9.8%)	
Educational attainment		
University (MSc/BSc)	64 (2.3%)	
Higher vocational education (BSc)	268 (9.7%)	
Middle vocational education	293 (10.6%)	
High school/secondary education	1555 (56.0%)	
<High school level	268 (9.7%)	

a For all PROMs except the EQ5D a lower score is better. For the EQ5D a higher score is better due to the way the total score was calculated.

### 3.3 Importance ratings

Although most PROM questions were considered important, some were deemed more or less important than others (Table 2); 77.7% of all patients undergoing hip surgery rated being able to run as not very important or unimportant. Pivoting or twisting on a loaded leg was rated not very important or unimportant by 22.6% of patients who underwent hip surgery. For knee surgery, being able to pivot or twist on the injured leg was considered not very important or unimportant by 15.6% of the patients. Patients who underwent knee surgery were even less interested in being able to kneel (32.7%) or squat (39.6%). A reduction in pain, and especially in pain during rest, was considered very important by both hip (68.2%) and knee (66.5%) surgery patients.

**Table 2:** Frequencies of the importance ratings (N=2776)

	N (%)	Completely unimportant (%)	Not very important (%)	Important (%)	Very important (%)
HOOS‐PS item 1: Descending stairs	1389 (95.3%)	3.5	6.6	49.2	40.7
HOOS‐PS item 2: Getting in/out of bath or shower	1391 (95.4%)	1.5	2.4	49.3	46.7
HOOS‐PS item 3: Sitting	1393 (95.5%)	1.2	1.1	40.4	57.3
HOOS‐PS item 4: Running	1329 (91.2%)	31.8	45.9	15.2	7.1
HOOS‐PS item 5: Twisting/pivoting on your loaded leg	1357 (93.1%)	4.5	18.1	54.5	22.9
NRS pain in rest (hip)	1390 (95.3%)	1.7	0.9	29.2	68.2
NRS pain during use (hip)	1397 (95.8%)	1.6	1.1	37.4	59.9
KOOS‐PS item 1: Rising from bed	1274 (96.6%)	1.6	3.4	54.6	40.5
KOOS‐PS item 2: Putting on socks/stockings	1278 (97.0%)	2.0	3.1	58.9	36.1
KOOS‐PS item 3: Rising from sitting	1272 (96.5%)	1.3	1.5	55.9	41.3
KOOS‐PS item 4: Bending to the floor	1276 (96.8%)	1.5	4.7	61.2	32.6
KOOS‐PS item 5: Twisting/pivoting on your injured knee	1269 (96.3%)	1.6	14.0	57.8	26.6
KOOS‐PS item 6: Kneeling	1254 (95.1%)	5.3	27.4	46.1	21.2
KOOS‐PS item 7: Squatting	1242 (94.2%)	6.7	32.9	41.1	19.4
NRS pain in rest (knee)	1272 (96.5%)	1.2	1.2	31.1	66.5
NRS pain during use (knee)	1273 (96.6%)	0.9	1.2	36.8	61.1
EQ5D item 1: Mobility	2686 (96.8%)	0.8	0.5	34.2	64.5
EQ5D item 2: Self‐care	2687 (96.8%)	1.5	0.7	36.2	61.6
EQ5D item 3: Usual activities	2659 (95.8%)	1.0	1.7	44.2	53.1
EQ5D item 4: Pain/discomfort	2664 (96.0%)	0.8	1.0	36.6	61.6
EQ5D item 5: Anxiety/depression	2651 (95.5%)	1.7	1.9	34.5	61.9

### 3.4 Factors which influence what patients consider important

Many factors determine the level of importance patients allocate towards the PROM items. For the HOOS‐PS items, the level of importance mainly depended upon age, overall health, and to a lesser extend the reason for surgery (Table 3). In contrast, the importance of the KOOS‐PS items was related to the reason for surgery, sex, and overall psychological health (Table 4). Importance ratings for the EQ5D were related to age, sex, overall health and overall psychological health (Table 5). Patients who chose to have surgery to improve their functioning allocated more importance towards two of the five HOOS‐PS items and four of seven items of the KOOS‐PS than patients who wished for pain relief. Younger patients considered four of five EQ5D items and three HOOS‐PS items more important than older patients. Younger, healthier hip surgery patients and patients who wanted to improve their functioning placed a higher value on more demanding movements such as descending stairs and running than other patients. Psychologically healthier patients considered more items from the KOOS‐PS (four of seven items), and the EQ5D (four of five items) important. Overall healthier patients considered more items of the HOOS‐PS (four items) and the EQ5D (five items) important. Women considered many EQ‐5D items (three of five items) and KOOS‐PS items (five of seven items) more important than men. Finally, hip surgery patients who were younger rated a reduction in both pain during rest and pain during use as more important (Table 6).

**Table 3:** Factors related to the importance ratings corresponding to the items of the HOOS‐PS

	HOOS‐PS rating item 1 (N=1061)			HOOS‐PS rating item 2 (N=1071)			HOOS‐PS rating item 3 (N=1080)			HOOS‐PS rating item 4 (N=954)			HOOS‐PS rating item 5 N=1034)		
	β	95% CI	*P*	β	95% CI	*P*	β	95% CI	*P*	β	95% CI	*P*	β	95% CI	*P*
HOOS‐PS pre‐score item 1^a^	−.04	−.07 to .01	.18												
HOOS‐PS pre‐score item 2^a^				.07	.00‐.06	.03									
HOOS‐PS pre‐score item 3^a^							.09	.01‐.07	.00						
HOOS‐PS pre‐score item 4^a^										−.13	−.20 to .07	.00			
HOOS‐PS pre‐score item 5^a^													.02	−.03 to .05	.60
Reason for surgery: Function (ref)															
Pain	−.10	−.23 to .05	.00	−.08	−.17 to .02	.02	−.05	−.13 to .02	.13	−.08	−.25 to .03	.01	−.08	−.21 to .02	.02
Unable to choose between reasons	.02	−.09 to .18	.52	.02	−.08 to .16	.54	.07	.01‐.23	.03	.01	−.14 to .20	.76	.01	−.12 to .18	.66
Complications	−.05	−.01 to .00	.11	−.06	−.00 to .00	.06	−.03	−.00 to .00	.29	.00	−.00 to .00	.95	−.04	−.00 to .00	.23
Age	−.19	−.02 to .01	.00	−.03	−.01 to .00	.35	.01	−.00 to .00	.79	−.14	−.02 to .01	.00	−.17	−.02 to .01	.00
Sex (female)	−.05	−.16 to .02	.13	.01	−.06 to .10	.69	.03	−.03 to .11	.29	−.11	−.31 to .08	.00	−.04	−.17 to .03	.18
Education: Lower to middle vocational education (ref)															
>High school level	.03	−.10 to .24	.41	−.03	−.22 to .08	.38	−.08	−.31 to .03	.02	−.09	−.55 to .07	.01	.03	−.12 to .25	.49
High school/secondary education	.10	.03‐.25	.02	−.00	−.10 to .09	.94	−.07	−.17 to .01	.09	−.01	−.16 to .11	.74	.03	−.07 to .17	.44
Higher vocational education (BSc)	.11	.09‐.39	.00	.03	−.07 to .20	.38	.02	−.09 to .16	.57	.07	.00‐.37	.05	.03	−.09 to .24	.37
University (BSc/MSc)	.04	−.10 to .41	.22	.04	−.09 to .36	.24	.03	−.10 to .31	.31	−.01	−.38 to .26	.71	.03	−.14 to .42	.32
Overall health^b^	−.11	−.15 to .03	.00	−.07	−.10 to .00	.06	−.10	−.12 to .02	.01	−.17	−.25 to .11	.00	−.09	−.14 to .01	.02
Overall psychological health^b^	−.04	−.08 to .02	.20	−.09	−.10 to .01	.01	−.08	−.09 to .01	.02	−.00	−.06 to .06	.97	−.07	−.11 to .00	.04
Number of days after surgery	.02	.00‐.00	.41	−.02	−.00 to .00	.48	.03	.00‐.00	.30	.06	.00‐.00	.06	−.06	−.00 to .00	.04

a Analyses were controlled for the PROM pre‐score. Although all pre‐scores are included in the table, only the pre‐score corresponding to the dependent variable was included.

b A lower score indicates better experienced health.

**Table 4:** Factors related to the importance ratings corresponding to items of the KOOS‐PS

	KOOS‐PS rating item 1 (N=1045)			KOOS‐PS rating item 2 (N=1031)			KOOS‐PS rating item 3 (N=1022)			KOOS‐PS rating item 4 (N=1031)			KOOS‐PS rating item 5 (N=1024)			KOOS‐PS rating item 6 (N=1018)			KOOS‐PS rating item 7 (N=1005)		
	β	95% CI	*P*	β	95% CI	*P*	β	95% CI	*P*	β	95% CI	*P*	β	95% CI	*P*	β	95% CI	*P*	β	95% CI	*P*
KOOS‐PS pre‐score item 1^a^	.05	−.01 to .06	.11																		
KOOS‐PS pre‐score item 2^a^				.07	.00‐.06	.04															
KOOS‐PS pre‐score item 3^a^							.12	.03‐.08	.00												
KOOS‐PS pre‐score item 4^a^										.04	−.01 to .05	.23									
KOOS‐PS pre‐score item 5^a^													.01	−.03 to .04	.84						
KOOS‐PS pre‐score item 6^a^																−.06	−.09 to .00	.06			
KOOS‐PS pre‐score item 7^a^																			−.09	−.12 to .02	.01
Reason for surgery: Function (ref)																					
Pain	−.05	−.14 to .02	.15	−.07	−.17 to .01	.03	−.04	−.13 to .03	.19	−.09	−.20 to .04	.00	−.10	−.23 to .05	.00	−.09	−.27 to .05	.01	−.10	−.28 to .06	.00
Unable to choose between reasons	−.03	−.17 to .08	.44	.01	−.11 to .13	.87	−.02	−.14 to .09	.65	−.03	−.19 to .06	.32	.02	−.11 to .17	.65	.04	−.08 to .26	.29	−.00	−.17 to .17	.98
Complications	.02	−.00 to .00	.43	.03	−.00 to .00	.28	.03	−.00 to .00	.37	.03	−.00 to .00	.32	−.00	−.00 to .00	.92	−.02	−.00 to .00	.48	−.06	−.01 to .00	.06
Age	.09	.00‐.01	.01	.09	.00‐.01	.01	.06	.00‐.01	.05	.00	−.00 to .01	.94	.01	−.01 to .01	.71	.01	−.01 to .01	.75	.01	−.01 to .01	.76
Sex (female)	.08	.02‐.17	.02	.10	.05‐.20	.00	.09	.03‐.18	.01	−.11	.05‐.20	.00	−.11	−.24 to .06	.00	−.09	−.26 to .05	.00	−.02	−.14 to .08	.54
Education: Lower to middle vocational education (ref)																					
>High school level	−.05	−.24 to .05	.21	−.05	−.26 to .04	.15	−.08	−.31 to .03	.02	−.01	−.21 to .08	.76	−.02	−.19 to .14	.70	.02	−.15 to .25	.64	.03	−.13 to .28	.48
High school/secondary education	−.02	−.12 to .06	.54	−.01	−.11 to .08	.76	−.06	−.15 to .03	.16	.02	−.10 to .09	.54	.02	−.07 to .14	.60	.05	−.04 to .21	.20	.06	−.03 to .24	.11
Higher vocational education (BSc)	.01	−.13 to .16	.82	−.01	−.12 to .17	.77	.04	−.07 to .21	.30	.02	−.04 to .25	.61	.01	−.12 to .21	.77	−.01	−.21 to .18	.88	−.03	−.29 to .12	.40
University (BSc/MSc)	.04	−.08 to .44	.18	.01	−.22 to .30	.75	.01	−.23 to .29	.82	−.01	−.16 to .35	.71	−.02	−.34 to .23	.49	.00	−.32 to .38	.86	−.02	−.50 to .24	.50
Overall health^b^	−.04	−.09 to .02	.23	−.03	−.08 to .03	.41	−.03	−.07 to .03	.40	.02	−.07 to .04	.67	.01	−.05 to .07	.86	−.02	−.09 to .05	.58	−.01	−.08 to .07	.84
Overall psychological health^b^	−.10	−.12 to .02	.00	−.10	−.12 to .02	.00	−.09	−.10 to .01	.02	−.09	−.13 to .04	.01	−.11	−.12 to .02	.00	.00	−.06 to .06	.96	−.01	−.07 to .06	.82
Number of days after surgery	−.02	−.00 to .00	.46	−.02	−.00 to .00	.64	−.02	−.00 to .00	.53	−.03	.00‐.00	.36	−.04	−.00 to .00	.24	.00	−.00 to .00	.97	−.02	−.00 to .00	.60

a Analyses were controlled for the PROM pre‐score. Although all pre‐scores are included in the table, only the pre‐score corresponding to the dependent variable was included.

b A lower score indicates better experienced health.

**Table 5:** Factors related to the importance ratings corresponding to the items of the EQ5D

	EQ5D rating item 1 (N=2091)			EQ5D rating item 2 (N=1982)			EQ5D rating item 3 (N=1978)			EQ5D rating item 4 (N=2015)			EQ5D rating item 5 (N=2007)		
	β	95% CI	*P*	β	95% CI	*P*	β	95% CI	*P*	β	95% CI	*P*	β	95% CI	*P*
EQ5D pre‐score item 1^a^	.02	−.05 to .10	.46												
EQ5D pre‐score item 2^a^				.02	−.03 to .07	.40									
EQ5D pre‐score item 3^a^							−.00	−.05 to .04	.93						
EQ5D pre‐score item 4^a^										.08	.04‐.12	.00			
EQ5D pre‐score item 5^a^													.04	−.01 to .08	.10
Reason for surgery: Function (ref)															
Pain	−.02	−.07 to .03	.42	.00	−.05 to .06	.95	−.06	−.12 to .01	.02	.08	.03‐.14	.00	.02	−.03 to .08	.44
Unable to choose between reasons	.02	−.03 to .11	.30	.04	−.01 to .15	.09	.03	−.02 to .14	.16	.07	.04‐.19	.00	.07	.04‐.21	.00
Complications	−.01	−.00 to .00	.58	.01	−.00 to .00	.66	−.03	−.00 to .00	.17	−.02	−.00 to .00	.50	.01	−.00 to .00	.69
Age	−.13	−.01 to .01	.00	−.07	−.01 to .00	.00	−.17	−.01 to .01	.00	−.08	−.01 to .00	.00	−.01	−.00 to .00	.55
Sex (female)	.04	−.00 to .09	.06	.10	.06‐.17	.00	.07	.03‐.13	.00	.01	−.04 to .06	.56	.07	.03‐.15	.00
Education: Lower to middle vocational education (ref)															
>High school level	−.04	−.16 to .02	.11	−.01	−.13 to .08	.68	−.04	−.17 to .03	.16	.00	−.09 to .10	.95	−.07	−.25 to .03	.01
High school/secondary education	.02	−.03 to .08	.40	.02	−.04 to .09	.51	.00	−.06 to .07	.95	.02	−.04 to .08	.56	−.03	−.10 to .04	.34
Higher vocational education (BSc)	.04	−.02 to .14	.16	.07	.03‐.21	.01	.06	.01‐.19	.03	.04	−.02 to .15	.15	.00	−.09 to .11	.89
University (BSc/MSc)	.00	−.14 to .15	.98	.02	−.10 to .22	.48	.01	−.11 to .21	.56	−.05	−.31 to .00	.05	−.03	−.27 to .07	.26
Overall health^b^	−.08	−.08 to .02	.00	−.09	−.10 to .02	.00	−.09	−.10 to .03	.00	−.11	−.11 to .04	.00	−.08	−.10 to .02	.00
Overall psychological health^b^	−.11	−.09 to .03	.00	−.11	−.10 to .03	.00	−.11	−.10 to .04	.00	−.05	−.06 to .00	.04	−.11	−.10 to .04	.00
Number of days after surgery	−.01	.00‐.00	.78	.00	.00‐.00	.94	.02	.00‐.00	.35	.02	.00‐.00	.30	.03	.00‐.00	.24
Surgery type (Knee)	−.03	−.07 to .02	.21	−.03	−.09 to .02	.18	−.05	−.10 to .01	.03	−.03	−.07 to .02	.24	−.01	−.07 to .04	.59

a Analyses were controlled for the PROM pre‐score. Although all pre‐scores are included in the table, only the pre‐score corresponding to the dependent variable was included.

b A lower score indicates better experienced health.

**Table 6:** Factors related to the importance rating corresponding to the NRS pain items

	NRS pain in rest (hip) (N=1070)			NRS pain during use (hip) (N=1078)			NRS pain in rest (knee) (N=1021)			NRS pain during use (knee) (N=1024)		
	β	95% CI	*P*	β	95% CI	*P*	β	95% CI	*P*	β	95% CI	*P*
NRS pain in rest pre‐score^a^	.04	−.00 to .02	.16				.11	.01‐.04	.00			
NRS pain during use^a^				.07	.00‐.03	.03				.18	.03‐.06	.00
Reason for surgery: Function (ref)												
Pain	.05	−.01 to .14	.10	.03	−.05 to .11	.43	.03	−.04 to .11	.39	−.03	−.11 to .05	.33
Unable to choose between reasons	.03	−.06 to .17	.35	.02	−.08 to .16	.47	.04	−.05 to .18	.28	.01	−.11 to .12	.87
Complications	−.03	−.00 to .00	.37	−.03	−.00 to .00	.37	−.03	−.00 to .00	.43	−.03	−.00 to .00	.33
Age	−.13	−.01 to .00	.00	−.12	−.01 to .00	.00	−.05	−.01 to .00	.17	−.04	−.01 to .00	.25
Sex (female)	.06	−.00 to .15	.06	.06	.00‐.16	.05	.00	−.07 to .08	.89	.04	−.03 to .12	.20
Education: Lower to middle vocational education (ref)												
>High school level	−.02	−.23 to .05	.45	−.02	−.19 to .10	.48	−.05	−.24 to .04	.17	−.04	−.22 to .05	.22
High school/secondary education	−.06	−.16 to .03	.17	−.08	−.19 to .00	.05	−.01	−.10 to .08	.88	−.02	−.11 to .07	.70
Higher vocational education (BSc)	.01	−.10 to .15	.71	.00	−.13 to .14	.96	.06	−.03 to .25	.11	−.00	−.14 to .13	.93
University (BSc/MSc)	−.00	−.22 to .21	.99	−.04	−.36 to .09	.23	.02	−.17 to .31	.55	−.01	−.27 to .21	.79
Overall health^b^	−.07	−.10 to .00	.04	−.09	−.12 to .02	.01	−.02	−.07 to .03	.51	−.07	−.10 to .00	.05
Overall psychological health^b^	−.10	−.10 to .02	.01	−.08	−.09 to .01	.03	−.06	−.08 to .01	.12	−.02	−.05 to .03	.64
Number of days after surgery	−.02	−.00 to .00	.45	−.02	−.00 to .00	.48	−.05	−.00 to .00	.14	−.05	−.00 to .00	.11

a Analyses were controlled for the PROM pre‐score. Although all pre‐scores are included in the table, only the pre‐score corresponding to the dependent variable was included.

b A lower score indicates better experienced health.

## 4 DISCUSSION

The present study's aim was to give insight into the relevance of a few well‐known PROMs from a patient's perspective and the possible differences in preferences between patients. Even though the PROMs included in this study were developed without patient involvement, patients considered most items of the KOOS‐PS, HOOS‐PS, EQ‐5D and NRS pain important. However, there are certainly a few items included in the PROMs which reflect the outcomes patients prefer to achieve less well. Perhaps the most remarkable question which was rated unimportant by 77.7% of patients is the item “running,” which is part of the HOOS‐PS. Most hip replacement patients are well into old age, with an average age of 73 in this study. Running is therefore for most patients an unlikely activity. However, without a “not applicable” option available, patients are forced to choose an option. Other less essential functions were found in the KOOS‐PS, for example the item “squatting.” Items which do not measure what many patients consider important may impact the insight the PROM may offer into the patient's improvement^17^, ^39^, ^40^, ^41^ or may even keep patients from completing the questionnaire.^19^ If these items cannot be replaced by more relevant items, perhaps taking into account importance ratings can help ensure that the quality of care is measured through items which are relevant to or even achievable by patients.

This study also investigated whether certain factors related to the patient may influence which items of the PROMs patients consider important. Patient specific factors such as age, sex, general health, overall psychological health and the main reason for undergoing surgery were important factors in determining what patients considered important. Earlier research investigating the influence of demographic variables on the importance of aspects of patient‐centred care found similar results.^42^, ^43^ It appears that especially younger, healthier, female patients consider many aspects of both processes and outcomes of care important. Further research is needed to investigate why certain outcomes are more important to certain patient groups. Perhaps more importantly, patients, who were older, experienced overall bad health and psychological health or patients who indicated that their main reason for surgery was pain reduction, considered many items less important than other patients. As the PROMs are chosen for measuring outcomes that patients can expect from surgery, the question is whether treatment outcomes will match these patients’ preferences and whether patients will benefit optimally from surgery.

### 4.1 Limitations and strengths

The present findings should be regarded with some caution because of study limitations. First, this study used a retrospective post‐then‐pre design. Measuring the patient's outcomes after surgery may have influenced the accuracy and completeness of patients’ recall. Research, however, indicates that the impact of measuring afterwards instead of before and after is minimal.^44^ It may even improve the accuracy as no response shift takes place.^45^ Second, all patients who underwent surgery during the year 2014 were invited to participate, while the questionnaires were sent out all at once. This means that the time between surgery and questionnaire completion varies. Although analyses were controlled for the number of days after surgery, results may be influenced by a recall bias. Third, respondents were on average about a year younger than non‐respondents. As our results show that older patients consider many aspects of outcomes less important, inclusion of these non‐respondents would probably have led to greater variance in the importance ratings. Fourth, importance ratings were used because it is an uncomplicated method for gaining insight into patient preferences regarding aspects of PROMs.^26^ However, the disadvantages of using importance ratings are that it is possible for patients to rate every item as important^42^ and that the overall results tend to be skewed.^26^ As suggested by Sixma et al.,^26^ the skewness of the results was counteracted by having a greater variation in the dimension of important. Fifth, hospitals were selected based on the average number of patients and an even distribution of hip and knee surgery patients. Therefore, the results may not accurately reflect all hospitals.

An important strength of this study is that, although patients were not actively involved in the study, by adding importance ratings to a PROMs survey, this study was able to give some important insights into the relevance of PROMs from the patients' perspective. Additionally, the study also gave more insight into patients’ preferences regarding outcomes of hip and knee surgery. These results have important consequences for the use of PROMs during medical consultations and for measuring the value of health care. The results of this study are therefore a useful contribution towards the discussion regarding patient involvement in research, health care and medical practice.

### 4.2 Implications

Our results show that not all aspects of the PROMs are considered equally important. Furthermore, patients appear to differ in what they consider important. These results have several important implications for the use of PROMs. Firstly, as not all aspects of the PROMs are considered equally important, these PROMs may not optimally reflect improvement due to surgery from the patient's perspective. Besides aspects which may be less important, there may also be important aspects which are missing from the PROMs. The only way to establish this and create a PROM which truly reflects the patient's perspective is by involving patients throughout the development process. Fortunately, patient involvement is increasingly required by organizations such as the FDA.^46^

Secondly, when PROMs are used to measure quality of care it may be important to take into account the differences in preferences between patients. For example, surgery results could be made more representative of patients by weighing the results using importance ratings. This would among others highlight which aspects of care that are important to patients can be improved upon. Furthermore, taking into account patient preferences using a similar method as for case‐mix adjustment may be important for comparing hospitals. Neglecting differences in patients’ preferences while interpreting PROM results in this case may mean that the patient's health and, further down the line, health‐care providers could be judged on an outcome which is not relevant to patients. Taking into account individual differences by weighting PROM results is not a solution for the negligence of the patients’ perspective during PROM design. However, it may be a viable option to at least make sure that the results give an accurate view on how patients perceived the quality of their care of the measured outcomes that were included and do matter.

Thirdly, weighing PROM results as a kind of case‐mix adjustment may be useful to ensure that hospital performance is judged on outcome aspects which are relevant to patients. However, this may be less useful if there is no variation in importance ratings between hospitals, as this would have no effect on the PROM results. As hospital variance exists for many factors such as patient characteristics, preoperative health and outcomes,^47^, ^48^ further research may be needed to look at whether weighing PROM results impacts the order of hospitals when arranged according to performance.

Additionally, weighing results using importance ratings could be difficult due to several practical and even psychometric problems depending on how the constructs measured by the PROMs are viewed. If the construct is an aggregation of the separate dimensions, then it may be the case that the construct is no longer measured if some dimensions are taken out, or if dimensions are not aggregated in the correct fashion. For example, function is measured by several aspects of function. If you remove certain aspects, it is possible that you no longer fully cover the construct. The construct can also be viewed as a latent dimension, where scores on domains indicate where patients are positioned on the continuum of the latent dimension. In this case taking into account importance ratings may make determining where patients need to be positioned very complicated.^49^ For example, the easiest function is placed at the beginning of the continuum and the most difficult function at the end of the continuum. If a patient is able to use almost all functions, he or she will be placed near the end of the continuum. Weighing results will mean that some functions are taken into account more than others. This would make it impossible to place the patient on the continuum. Further discussion on whether and how importance ratings should be integrated into PROM results is needed. However, if the construct is indeed seen as a latent dimension, a suggestion is to not weigh the results. Instead, one could adjust the continuum to ensure that items at the end of the spectrum still represent aspects of the construct patients and their physicians feel are relevant and achievable. For example, in the case of many hip surgery patients being able to run is both unimportant and unachievable. For these patients adjusting the continuum would mean that the most demanding outcome becomes a more attainable outcome such as twisting on a loaded leg instead of running. This way patients are more likely to complete the questionnaire^19^ and physicians are not only judged on outcomes which may be a better representation of what is relevant to patients, but they are also judged on outcomes which are actually achievable for a specific patient.

Fifth, variation in preferences between patients is also relevant for patient‐provider communication. Patients are becoming more actively involved in their treatment, and awareness of patient's values, needs and preferences is important to individualize care^8^ as part of a person‐centred approach.^22^ For the elective surgery procedures which were investigated in this study, there are several suitable treatment options besides surgery.^50^ Taking into account patient's preferences may result in patients receiving the most appropriate treatment, which is both better for the patient and for the value of our health care.^51^ As this study indicates that patients’ preferences do vary, it is important that patients and their health‐care providers discuss and take into account patient preferences and the benefits and risks of the treatment options to come to a well‐informed decision.^42^, ^52^ Coming to a well‐informed treatment decision may be especially important for the patients who considered several outcomes of joint replacement less important than other patients (older patients, male patients, patients who experience overall bad health and psychological health and patients who mainly want to decrease their pain level). For these patients, other treatment options may be able to offer outcomes which are of more importance to these patients without having to undergo surgery.

## 5 CONCLUSION

Although many items from the PROMs included in this study were important to patients, not all aspects are equally important. Preferences also appear to differ between patients. Especially older, male patients, patients who experienced overall bad health and patients who experienced bad psychological health considered many aspects of the PROMs less important than other patients. These results have important consequences for the use of PROMs during medical consultations and for measuring the value of health care. The differences in preferences between patient groups indicate that it is important for health‐care professionals to explore patients’ preferences and discuss which treatment options best fit the patient's preferences. Furthermore, as PROMs are used to establish the value of health care, the variations in the importance levels may need to be taken into account to ensure that PROMs give an accurate view on how patients perceived the quality of their care. Further research is needed to investigate whether and how variations in importance can be integrated into PROM results.

## References

[1] Custers T , Klazinga NS , Brown AD . Increasing performance of health care services within economic constraints: working towards improved incentive structures. Z Arztl Fortbild Qualitatssich. 2007;101:381‐388. 17902405 10.1016/j.zgesun.2007.05.004

[2] Custers T , Arah OA , Klazinga NS . Is there a business case for quality in The Netherlands? A critical analysis of the recent reforms of the health care system. Health Policy. 2007;82:226‐239. 17070956 10.1016/j.healthpol.2006.09.005

[3] Black N . Patient reported outcome measures could help transform healthcare. BMJ. 2013;346:f167. 23358487 10.1136/bmj.f167

[4] Devlin NJ , Parkin D , Browne J . Patient‐reported outcome measures in the NHS: new methods for analysing and reporting EQ‐5D data. Health Econ. 2010;19:886‐905. 20623685 10.1002/hec.1608

[5] Larsson S , Lawyer P , Garellick G , Lindahl B , Lundström M . Use of 13 disease registries in 5 countries demonstrates the potential to use outcome data to improve health care's value. Health Aff. 2011;31:220‐227. https://doi.org/10.1377/hlthaff. 2011.0762. 10.1377/hlthaff.2011.0762 22155485

[6] VanLare JM , Conway PH . Value‐based purchasing—national programs to move from volume to value. N Engl J Med. 2012;367:292‐295. 22830460 10.1056/NEJMp1204939

[7] http://www.cms.gov/Regulations-andGuidance/Legislation/EHRIncentivePrograms/Downloads/EP_MeasuresTable_Posting_CQMs.pdf. Accessed 31 December 2014.

[8] Ekman I , Swedberg K , Taft C , et al. Person‐centered care—ready for prime time. Eur J Cardiovasc Nurs. 2011;10:248‐251. 21764386 10.1016/j.ejcnurse.2011.06.008

[9] Porter ME , Teisberg EO . Redefining Health Care: Creating Value‐Based Competition on Results. Massachusetts, USA: Harvard Business Press; 2006.

[10] Porter ME . A strategy for health care reform—toward a value‐based system. N Engl J Med. 2009;361:109‐112. 19494209 10.1056/NEJMp0904131

[11] Porter ME . What is value in health care? N Engl J Med. 2010;363:2477‐2481. 21142528 10.1056/NEJMp1011024

[12] Porter ME . Defining and introducing value in health care In: McClellan MB, Michael McGinnis J, Nabel EG, Olsen LM, eds. Evidence‐based medicine and the changing nature of health care. Washington, D.C.: The National Academies Press; 2008:61.

[13] Nelson EC , Eftimovska E , Lind C , Hager A , Wasson JH , Lindblad S . Patient reported outcome measures in practice. BMJ. 2015;350:g7818. 25670183 10.1136/bmj.g7818

[14] Trujols J , Portella MJ , Iraurgi I , Campins MJ , Siñol N , Cobos J . Patient‐reported outcome measures: are they patient‐generated, patient‐centred or patient‐valued? J Ment Health. 2013;22:555‐562. 23323928 10.3109/09638237.2012.734653

[15] Bredart A , Marrel A , Abetz‐Webb L , Lasch K , Acquadro C . Interviewing to develop Patient‐Reported Outcome (PRO) measures for clinical research: eliciting patients’ experience. Health Qual Life Outcomes. 2014;12:15. 24499454 10.1186/1477-7525-12-15 PMC3933509

[16] Wu AW , Snyder C , Clancy CM , Steinwachs DM . Adding the patient perspective to comparative effectiveness research. Health Aff. 2010;29:1863‐1871. 10.1377/hlthaff.2010.0660 20921487

[17] Kirwan JR , Fries JF , Hewlett S , Osborne RH . Patient perspective: choosing or developing instruments. J Rheumatol. 2011;38:1716‐1719. 21807791 10.3899/jrheum.110390

[18] Staniszewska S . Patient and public involvement in health services and health research: a brief overview of evidence, policy and activity. J Res Nurs. 2009;14:295‐298.

[19] Meadows KA . Patient‐reported outcome measures: an overview. Br J Community Nurs. 2011;16:146‐151. 21378658 10.12968/bjcn.2011.16.3.146

[20] Hibbard JH , Stockard J , Tusler M . Does publicizing hospital performance stimulate quality improvement efforts? Health Aff. 2003;22:84‐94. 10.1377/hlthaff.22.2.84 12674410

[21] Wiering B , Boer D , Delnoij D . Patient involvement in the development of patient‐reported outcome measures: a scoping review. Health Expect. 2017;20:11‐23. 26889874 10.1111/hex.12442 PMC5217930

[22] Wolf A . Person‐centred Care: Possibilities, Barriers and Effects in Hospitalised Patients. Gothenburg, Sweden; 2012.

[23] McClellan MB , McGinnis JM , Nabel EG , Olsen LM . Evidence‐Based Medicine and the Changing Nature of Health Care: Meeting Summary (IOM Roundtable on Evidence‐Based Medicine). Washington, D.C.: National Academies Press; 2008. 21391346

[24] Neumann PJ , Goldie SJ , Weinstein MC . Preference‐based measures in economic evaluation in health care. Annu Rev Public Health. 2000;21:587‐611. 10884966 10.1146/annurev.publhealth.21.1.587

[25] Suarez‐Almazor M , Kendall C , Johnson J , Skeith K , Vincent D . Use of health status measures in patients with low back pain in clinical settings. Comparison of specific, generic and preference‐based instruments. Rheumatology. 2000;39:783‐790. 10908699 10.1093/rheumatology/39.7.783

[26] Sixma HJ , Kerssens JJ , Campen C , Peters L . Quality of care from the patients’ perspective: from theoretical concept to a new measuring instrument. Health Expect. 1998;1:82‐95. 11281863 10.1046/j.1369-6513.1998.00004.x PMC5139902

[27] http://www.stichtingmiletus.nl. Accessed 20 December 2016.

[28] Davis A , Perruccio A , Canizares M , et al. The development of a short measure of physical function for hip OA HOOS‐Physical Function Shortform (HOOS‐PS): an OARSI/OMERACT initiative. Osteoarthritis Cartilage. 2008;16:551‐559. 18296074 10.1016/j.joca.2007.12.016

[29] Perruccio AV , Lohmander LS , Canizares M , et al. The development of a short measure of physical function for knee OA KOOS‐Physical Function Shortform (KOOS‐PS)–an OARSI/OMERACT initiative. Osteoarthritis Cartilage. 2008;16:542‐550. 18294869 10.1016/j.joca.2007.12.014

[30] Rabin R , Charro F . EQ‐SD: a measure of health status from the EuroQol Group. Ann Med. 2001;33:337‐343. 11491192 10.3109/07853890109002087

[31] Hawker GA , Mian S , Kendzerska T , French M . Measures of adult pain: Visual Analog Scale for Pain (VAS Pain), Numeric Rating Scale for Pain (NRS PAIN), McGill Pain Questionnaire (MPQ), Short‐Form McGill Pain Questionnaire (SF‐MPQ), Chronic Pain Grade Scale (CPGS), Short Form‐36 Bodily Pain Scale (SF‐36 BPS), and Measure of Intermittent and Constant Osteoarthritis Pain (ICOAP). Arthritis Care Res. 2011;63:S240‐S252. 10.1002/acr.20543 22588748

[32] Roos EM , Roos HP , Lohmander LS , Ekdahl C , Beynnon BD . Knee injury and osteoarthritis outcome score (KOOS)—development of a self‐administered outcome measure. J Orthop Sports Phys Ther. 1998;28:88‐96. 9699158 10.2519/jospt.1998.28.2.88

[33] Nilsdotter AK , Lohmander LS , Klässbo M , Roos EM . Hip disability and osteoarthritis outcome score (HOOS)–validity and responsiveness in total hip replacement. BMC Musculoskelet Disord. 2003;4:1. 12777182 10.1186/1471-2474-4-10 PMC161815

[34] Davis AM , Perruccio AV , Canizares M , et al. Comparative, validity and responsiveness of the HOOS‐PS and KOOS‐PS to the WOMAC physical function subscale in total joint replacement for osteoarthritis. Osteoarthritis Cartilage. 2009;17:843‐847. 19215728 10.1016/j.joca.2009.01.005

[35] Peer MA , Lane J . The knee injury and osteoarthritis outcome score (KOOS): a review of its psychometric properties in people undergoing total knee arthroplasty. J Orthop Sports Phys Ther. 2013;43:20‐28. 23221356 10.2519/jospt.2013.4057

[36] De Groot IB , Favejee MM , Reijman M , Verhaar JA , Terwee CB . The Dutch version of the knee injury and osteoarthritis outcome score: a validation study. Health Qual Life Outcomes. 2008;6:1. 18302729 10.1186/1477-7525-6-16 PMC2289810

[37] De Groot I , Reijman M , Terwee C , et al. Validation of the Dutch version of the hip disability and osteoarthritis outcome score. Osteoarthritis Cartilage. 2007;15:104‐109. 16890460 10.1016/j.joca.2006.06.014

[38] Corp. I . IBM SPSS Statistics for Windows, Version 22.0. Armonk, NY: IBM Corp; 2013.

[39] McKenna SP . Measuring patient‐reported outcomes: moving beyond misplaced common sense to hard science. BMC Med. 2011;9:86. 21756344 10.1186/1741-7015-9-86 PMC3170214

[40] Paterson C . Seeking the patient's perspective: a qualitative assessment of EuroQol, COOP‐WONCA charts and MYMOP. Qual Life Res. 2004;13:871‐881. 15233501 10.1023/B:QURE.0000025586.51955.78

[41] Staniszewska S , Adebajo A , Barber R , et al. Developing the evidence base of patient and public involvement in health and social care research: the case for measuring impact. Int J Consum Stud. 2011;35:628‐632.

[42] de Boer D , Delnoij D , Rademakers J . The importance of patient‐centered care for various patient groups. Patient Educ Couns. 2013;90:405‐410. 22079211 10.1016/j.pec.2011.10.002

[43] Krupat E , Bell RA , Kravitz RL , Thom D , Azari R . When physicians and patients think alike: patient‐centered beliefs and their impact on satisfaction and trust. (Original Research). J Fam Pract. 2001;50:1057‐1063. 11742607

[44] Swindle S , Baker SS , Auld GW . Operation frontline: assessment of longer‐term curriculum effectiveness, evaluation strategies, and follow‐up methods. J Nutr Educ Behav. 2007;39:205‐213. 17606246 10.1016/j.jneb.2007.03.003

[45] Rohs FR , Langone CA , Coleman RK . Response shift bias: a problem in evaluating nutrition training using self‐report measures. J Nutr Educ. 2001;33:165‐170. 11953233 10.1016/s1499-4046(06)60187-5

[46] Food and Drug Administration US . Guidance for Industry Patient‐Related Outcome Measures: Use in Medical Product Development to Support Labeling Claims. Washington: US Department of Health and Human Services Food and Drug Administration; 2009.

[47] Silber JH , Rosenbaum PR , Ross RN . Comparing the contributions of groups of predictors: which outcomes vary with hospital rather than patient characteristics? J Am Stat Assoc. 1995;90:7‐18.

[48] Bozic KJ , Grosso LM , Lin Z , et al. Variation in hospital‐level risk‐standardized complication rates following elective primary total hip and knee arthroplasty. J Bone Joint Surg. 2014;96:640‐647. 24740660 10.2106/JBJS.L.01639

[49] Trauer T , Mackinnon A . Why are we weighting? The role of importance ratings in quality of life measurement. Qual Life Res. 2001;10:579‐585. 11822791 10.1023/a:1013159414364

[50] Jüni P , Reichenbach S , Dieppe P . Osteoarthritis: rational approach to treating the individual. Best Pract Res Clin Rheumatol. 2006;20:721‐740. 16979535 10.1016/j.berh.2006.05.002

[51] Epstein RM , Fiscella K , Lesser CS , Stange KC . Why the nation needs a policy push on patient‐centered health care. Health Aff. 2010;29:1489‐1495. 10.1377/hlthaff.2009.0888 20679652

[52] Dieppe P , Lim K , Lohmander S . Who should have knee joint replacement surgery for osteoarthritis? Int J Rheum Dis. 2011;14:175‐180. 21518317 10.1111/j.1756-185X.2011.01611.x

